# Appraising Important Medical Literature Biases: Uncorrected Statistical Mistakes and Conflicts of Interest

**DOI:** 10.3389/fmed.2022.925643

**Published:** 2022-07-22

**Authors:** Frank A. Orlando, Karyn M. Governale, Irene M. Estores

**Affiliations:** ^1^Department of Community Health and Family Medicine, University of Florida College of Medicine, Gainesville, FL, United States; ^2^Department of Physical Medicine and Rehabilitation, University of Florida College of Medicine, Gainesville, FL, United States

**Keywords:** errata, erratum, conflict of interest, data sharing, transparency, COVID

## Introduction

Evidence-based practice requires critically reading the medical literature on a regular basis. This had never been as important as during the height of the COVID-19 pandemic when science was often updated several times per day and preprints needed to be deciphered by clinicians on the fly. Scientific journals have peer-review processes of varying degrees of rigor designed not only to ensure high quality studies become science, but also to prevent mistakes of all shapes and sizes. Evident by the number of retracted COVID-19 articles (1), some feel this process was somewhat curtailed at the onset of COVID-19 as science was changing so quickly that sound publishing processes needed to find ways to keep up (2). When significant errors occur, which they inevitably do, honest or otherwise, readers of the medical literature need to be keen enough to catch these in print. Scoping or systematic reviews may not always be able to do it for us, and some errors may just never get published errata. Here we review two distinct examples how critically reading the literature can reveal a statistical mistake or bias that could change our interpretation of a study's stated conclusions, thereby potentially affecting our practice of medicine.

### Statistical Error Casts Doubt on Safety of a Controversial Treatment

Most would agree that publishers have an academic and ethical obligation to investigate and publish errata, expressions of concern, or article retractions when appropriate. We endeavored to respect this process when we came across what appears to be an important oversight in a cardiovascular practice guideline article while preparing our now published review, “Functional Medicine: Focusing on Imbalances in Core Metabolic Processes” (see page 487) (3). We share our experience contacting the authors, journal editorial staff, and publisher in an attempt to correct this error, especially since it puts a controversial functional medicine practice in a negative light by mistakenly emphasizing safety risk.

The practice guideline article erroneously mentions that more than 50% of the 18% of participants lost to follow up in the Trial to Assess Chelation Therapy (TACT) were from the EDTA chelation group (4), thus we began the process of requesting an erratum. The journal editorial staff instructed us to send this erratum request to the publisher. We sent the article PDF with highlights of the error and attached both the TACT article and its supplement with highlights showing where they documented that more than 50% of these withdraws were from *the placebo group* (TACT article page 1,244 and TACT supplement pages 11-12) (5). The reader may misinterpret that the unblinding concern is related to the chelation group rather than an overall comment about the large number of subjects lost to follow-up and a large percent from one group. We did not opine to the publisher that the article is biased because it erroneously attributes most of the patients lost to follow up to the treatment group and follows this with a statement about the potential risks of chelation therapy including death. We were clear, however, that despite this error, the practice guideline authors came to a similar conclusion about the efficacy of chelation in cardiac disease as the TACT authors. Furthermore, we stated our sole purpose in requesting an erratum was to allow proper and consistent interpretation of the TACT study results in the practice guideline article.

The publisher requested we submit our proposed edit, and we were informed that an addendum, correction, or erratum would not be produced for this guideline. We did not receive a response to our request for an explanation for this decision. Perhaps one could argue that their guideline articles are only corrected with the strictest of criteria; however, one relatively recent Erratum includes edits of table and section titles (6). When we consider why the erratum request might have been denied, we reflect on how a new medical therapy could potentially affect the market for the revascularization procedures recommended in the guideline article such as percutaneous coronary intervention (7).

### Data Sharing and a Conflict of Interest

Potential conflicts of interest need to be declared by each co-author because they can create biases that affect study design, data collection, data interpretation, and ultimately study conclusions. It is the readers' responsibility to routinely seek out such conflict of interest statements published within a scientific article as well as the author affiliations and organizations who performed and/or funded the study. Readers then need to interpret if and how these relationships could have affected study conclusions, which requires data transparency. Data transparency of clinical trials is especially important during public health emergencies like the COVID-19 pandemic where treatments and vaccinations were administered rapidly and widely (8).

Clinical trial data is typically shared through standard platforms in compliance with various policies and regulations, and it is widely accepted to be in the best interest of patients because the scientific process requires independent verification and replication of study results. Data sharing becomes even more critical when the company funding and performing a study is the very same company manufacturing the study drug, such as with the COVID-19 vaccine clinical trials (see links in reference 8, Table 1) (2, 8). For example, data integrity, regulatory oversight, the reliability of blinding, and the primary endpoint evaluation process have been heavily scrutinized in various COVID-19 vaccination trials (2, 8). These concerns have been amplified due to a relative lack in data transparency, transparency of regulatory decision making, and real-time transparency in COVID-19 vaccine clinical trials (8, 9). Without data sharing, these academic concerns cannot and have not been appropriately addressed by the scientific and medical community despite a global vaccine rollout (9).

## Discussion

Except for our personal experience, (3) we do not know the prevalence of article errors that are detected by readers but are knowingly left uncorrected by publishers despite errata requests. Errata can be errors that occur during manuscript processing like typos, misspellings, or minor errors in scientific logic, such as result interpretation, and are easily corrected because they do not affect the scientific integrity of the article itself (10). Journals can publish an “erratum notice” to correct such small, inadvertent errors by the authors or editorial staff. For possible problems with an article, journals can alert its readers by publishing an “expression of concern,” for example during an interim investigation, although this type of notice is not universal given many questions about standardization (11). In cases of important methodology errors, fraudulent data reporting, plagiarism, copyright infringement, duplicate publication, or other serious infractions, article retraction is warranted.

Publishers who have routine and sound procedures for erratum notices and article retraction (12) preserve the integrity of how we share and expand scientific knowledge so that we may provide the safest medical care with the best outcomes. When errata notices are done correctly, it demonstrates that the scientific process really works (13, 14). On the contrary, we are unsure the prevalence of erratum request denials in cases where a mistake exists that affects readers' comprehension of medical science. When more than a minor error is denied an erratum notice and an explanation (3), especially in a medical guideline article or what physicians consider a stalwart journal (4), it can put doubt in the scientific process and the basis of what we use to educate future and practicing physicians: the scientific literature. Our patient-care experience is that it also erodes the trust of those patients who entrust their care to physicians who base their clinical decisions on other's research conclusions.

In spite of the peer-review process, it is necessary to critically read the medical literature if we are to trust that study conclusions are worthy of treating our patients, no matter how high a journal's impact factor. Delving deep into an article means not only having a substantial understanding of how the study was performed, data analyzed, and results interpreted, but also the interests of those who performed and funded the study. When data is inaccessible to studies that already have major conflicts of interest, it brings into question the integrity of these studies' conclusions. Journals and their publishers should routinely correct errors readers discover in the medical literature, (13, 14) and balanced methods for creating greater data transparency (8) need to be implemented immediately to preserve the integrity of the scientific process (9). This will help ensure the evidence-based recommendations we give our patients have been thoroughly vetted by us and not just by those who may have a financial interest in mind.

## Author Contributions

FO came up with the idea. IE supervised. FO, KG, and IE wrote, edited, reviewed, and approved the article for submission. All authors contributed to the article and approved the submitted version.

## Funding

The University of Florida College of Medicine Department of Community Health and Family Medicine and the Bueno Educational Fund paid for this article's publishing fees.

## Conflict of Interest

The authors declare that the research was conducted in the absence of any commercial or financial relationships that could be construed as a potential conflict of interest.

## Publisher's Note

All claims expressed in this article are solely those of the authors and do not necessarily represent those of their affiliated organizations, or those of the publisher, the editors and the reviewers. Any product that may be evaluated in this article, or claim that may be made by its manufacturer, is not guaranteed or endorsed by the publisher.
